# Applying the Cloud Intelligent Classroom to the Music Curriculum Design of the Mental Health Education

**DOI:** 10.3389/fpsyg.2021.729213

**Published:** 2021-11-16

**Authors:** Yanan Liang, Shiyong Wu

**Affiliations:** ^1^Music College, Gannan Normal University, Ganzhou, China; ^2^School of Education, Cavite State University, Indang, Philippines

**Keywords:** deep learning, cloud intelligent classroom, college music majors, psychological education, course design

## Abstract

The cloud intelligent classroom, supported by modern technologies, is the main trend of curriculum design in the future. The purpose of this study is to explore the promotion and integration between digital technology and the curriculum design of mental health education in colleges and universities and realize their real value. First, the overall idea and practical value of the study are clarified after the relevant literature is reviewed. Second, the setting, the teaching methods, and the ideas of the cloud classrooms based on digital technology are elaborated in detail. Then, the final effect of mental health education in cloud intelligent classrooms is demonstrated and summarized after the teaching practice, a questionnaire survey, and the expert assessment. Finally, the research conclusions are drawn and the suggestions for constructing the cloud intelligent classrooms of mental health education are proposed based on the practice and surveys. The research is based on the reality of mental health education in colleges and universities, rational thinking, and action. While updating the means and methods of the curriculum design of the mental health education in the high school, it expands the connotation of cloud intelligent classroom and pursues the unity of “form” and “content.” The cloud intelligent classroom helps to improve the teaching quality of mental health education for the music majors in colleges and universities in the short term. Cloud intelligent classrooms can also help to achieve the curriculum design and teaching objectives.

## Introduction

At the beginning of 2005, the Ministry of Education and the WHO jointly formulate and promulgate the *opinions on further strengthening and improving the mental health education of college students*. It is proposed that the mental health education of college students should be effectively implemented and the important role of the mental health education of college students should be advocated. The Ministry of Education also promulgated the *basic requirements for the teaching of mental health education for college students* in 2011, which describes the detailed regulations on the curriculum design, teaching objectives, contents, and methods of mental health education in the colleges and universities. After more than 10 years of practice, the major colleges and universities pay more and more attention to the mental health education of the students and make new attempts to reform the curriculum design. However, the analysis of the survey results of relevant institutions reveals that the desire of students for the other relevant courses is rising. The curriculum design of mental health education is popular, which greatly affects the satisfaction of the students within the classroom and the curriculum design of the mental health education (Qian et al., 2018; Chen, 2019). The lack of the resources of curriculum construction, the backwardness of teaching materials, and the imperfect management mechanism hinders the development of mental health education. In addition, the original classroom courses are mainly based on the knowledge imparting, lacking practical application of knowledge due to the long-term neglect of the real needs of the students for the mental health education in the teaching practice. At the same time, there are also problems such as the single teaching methods and contents, which are all the key problems that need to be solved urgently in traditional mental health education (Wu and Wu, 2017).

**Figure 1:**
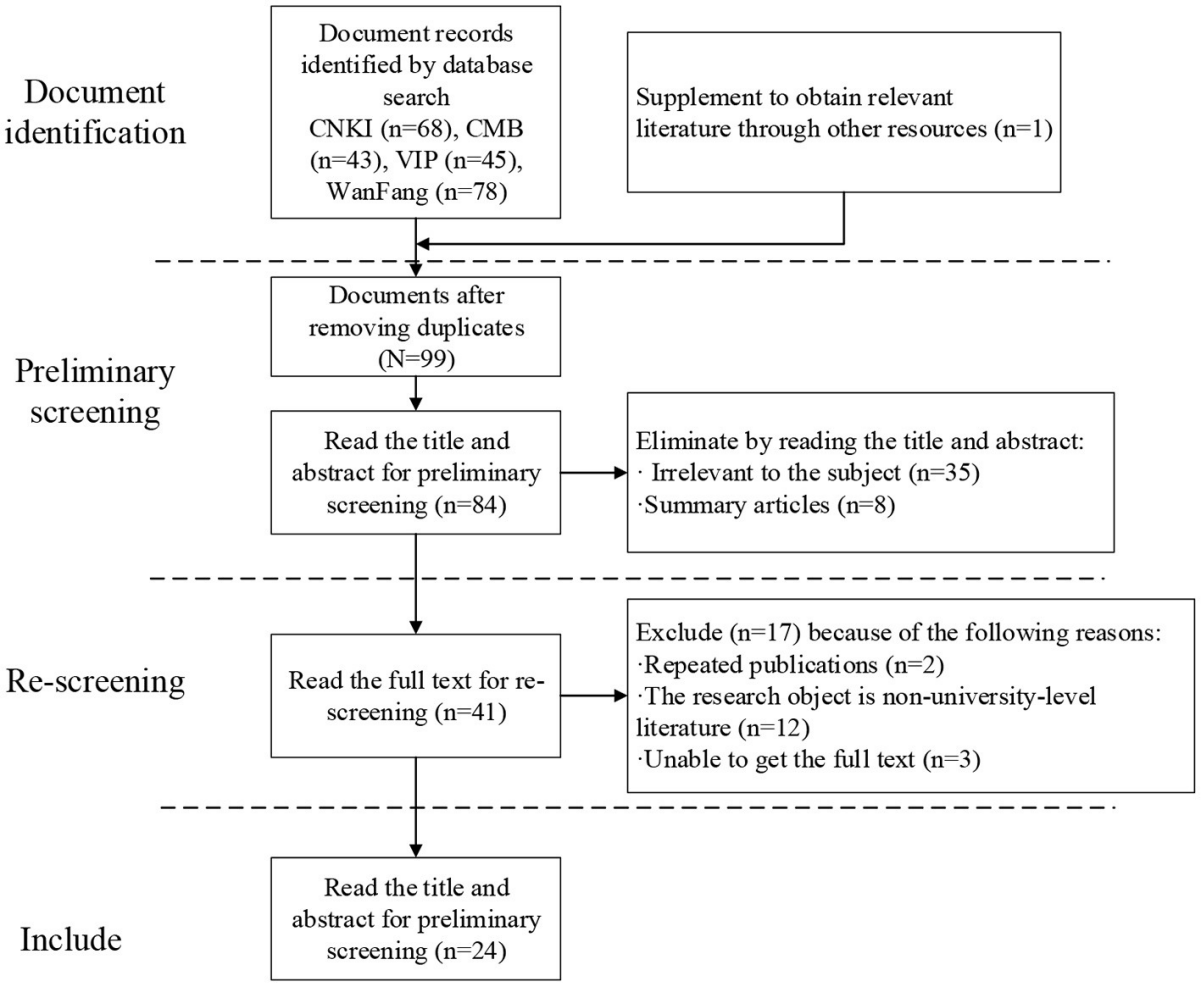
Flowchart of the literature screening.

At present, more attention is paid to the mental health education of the students majoring in music because the music majors have obvious differences in personality, emotion, temperament, and thinking. Therefore, the mental health education of the music majors should be carried out according to their mental states and the teaching modes of music majors, so that the mental health education can truly meet the characteristics and actual needs of music majors (Rogoza et al., 2018; Saha-Gupta and Song, 2019). As a new trend of curriculum construction and development at present, cloud intelligent classrooms have obvious advantages over the traditional classroom in the space and time constraints, which play an important role in promoting the development of mental health education for music majors. Therefore, this study is expected to explore the promotion of the curriculum construction of mental health education based on cloud intelligent classrooms, so that the practical value of the cloud intelligent classrooms can be fully embodied. According to the domestic and foreign literature, this study first clarifies the research ideas and their practical value (Wu and Song, 2019). Then, the ideas of cloud intelligent classrooms are put forward. Subsequently, the actual effect of cloud intelligent classrooms in mental health education is proved through practice, a questionnaire survey, and expert assessment. Finally, the research conclusions are drawn and the construction of cloud intelligent classrooms for mental health education is proposed. A total of 235 relevant pieces of literature is retrieved and 24 of them are referred according to the inclusion and exclusion criteria. The literature screening process mainly goes through four steps: document identification, preliminary screening, rescreening, and inclusion as shown in Figure 1.

Based on the summary of the domestic and foreign literature, previous studies on the mental health education curriculum mainly focus on the aspects of current situation investigation, concept analysis, function evaluation, and curriculum system construction. At present, the research on the school-based curriculum of mental health education in colleges and universities mostly focuses on the teaching design of a demonstration course, a hot topic, classroom records, after-school reflection, and so on (Wu et al., 2019; Kwet, 2020; Wu W. et al., 2020). At present, a comprehensive curriculum system covering the whole year has not been formed. At the same time, previous studies on cloud intelligent courses mainly focus on the new online teaching methods such as microcourses and massive open online courses (MOOCs). Under the framework of the university curriculum system, the cloud intelligent curriculum research focuses on improving the effect of classroom practice, more intuitive experimental demonstration, and more vivid presentation of the teaching materials (Aguilar and Holman, 2018; Yang et al., 2018; Zheng et al., 2018; Cebrian and Palau, 2020). The practice of the curriculum in the cloud intelligent room follows the basic curriculum design framework and technology only plays the role of “icing on the cake,” which fails to change the essence of the “giving and receiving” relationship between the teachers and students in the traditional classroom (Huang et al., 2019). After the domestic and foreign literature is reviewed, the research idea and its practical value are clarified. Then, the idea and measures of constructing the cloud intelligent classrooms are put forward according to the relevant theories of the cloud intelligent classrooms. Subsequently, the actual effect of cloud intelligent classrooms on mental health education is explored through practice, a questionnaire survey, and expert assessment. Finally, the research conclusions are drawn and the construction measures of the cloud intelligent classrooms for mental health education are proposed.

## Relevant Theories

### Related Theories of the Cloud Intelligent Classrooms

#### Architecture of the Teaching Model Based on the Cloud Intelligent Classrooms

**Figure 2:**
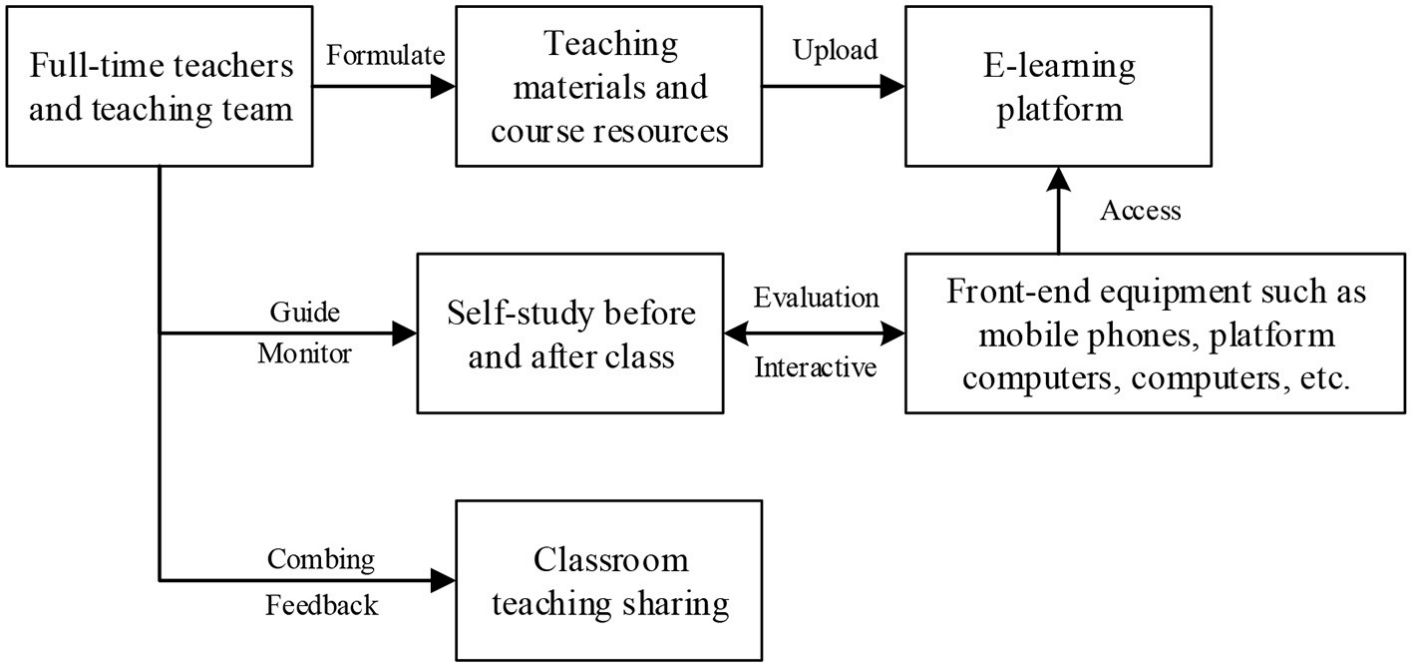
Architecture of the cloud intelligent classrooms.

Based on the cloud end, the traditional classroom is transferred to the network platform. At the same time, the roles of teachers and students change according to the intelligent teaching mode. The role of teachers is to provide learning resources and guide the students to learn the knowledge and skills. The role of students is “acting” mainly to complete autonomous learning, ask questions, and share results. Then, the teachers and students are evaluated in the cloud intelligent classrooms, fully highlighting the central position of students (Wu et al., 2019; Johnson et al., 2021; Yi et al., 2021). The teaching model based on cloud intelligent classrooms is shown in Figure 2.

#### Teaching Ideas and Measures of the Cloud Intelligent Classrooms

(1) Teaching ideas. The concept of cloud intelligent classrooms refers to the talent training goal that adheres to the outcome-based education (OBE) concept with the students as the main body and the learning results as the goal and the teaching of this course is continuously innovated as follows: (1) Formulate the teaching outline of the integration of production and education, (2) Determine the teaching objectives by grading and positioning and the expected learning results of the students, (3) Make the teaching plans and experimental arrangements, (4) The teaching plan is designed, (5) Teaching practice is carried out, (6) The expected learning results are evaluated, and (7) The curriculum evaluation is completed (Archer-Kuhn et al., 2020; Yue, 2021).

(2) Teaching measures. According to the teaching ideas of this course, the specific measures are carried out from the following aspects: (1) The government, colleges and universities, and enterprises should join the requirements and expectations of stakeholders that are fully considered to formulate the syllabus of the course and the key and difficult points of the content should be standardized, (2) According to the cognitive classification of educational objectives by Bloom, the teaching objectives are graded and the fit between the learning results of the students and teaching objectives is estimated (Kaihua, 2020; Paudel et al., 2020; Chavess and Taylor, 2021), (3) Make the teaching plans according to the syllabus including the arrangement of teaching time, the setting of experiment content, and the progress arrangement of the curriculum, (4) Based on the method of reverse design, namely, starting from the teaching goal of “cultivating the professional ability of the students,” the curriculum design is carried out according to the steps of the top-level drive, problem decomposition, and emphasis on cohesion (Krishnaswamy et al., 2019; Wu Y. J. et al., 2020; Yuan and Wu, 2020), (5) A variety of teaching stages should be integrated including preclass, afterclass, and online and offline teaching processes, (6) Learning effect of the students is evaluated before the teaching process, in the teaching process, and after the teaching process, and (7) The results of the course are summarized after the course is completed. In short, the cloud intelligent classroom teaching mode promotes and improves the implementation of deep learning.

## Research Methods

### Research Subjects and Tools

#### Research Ideas

**Figure 3:**
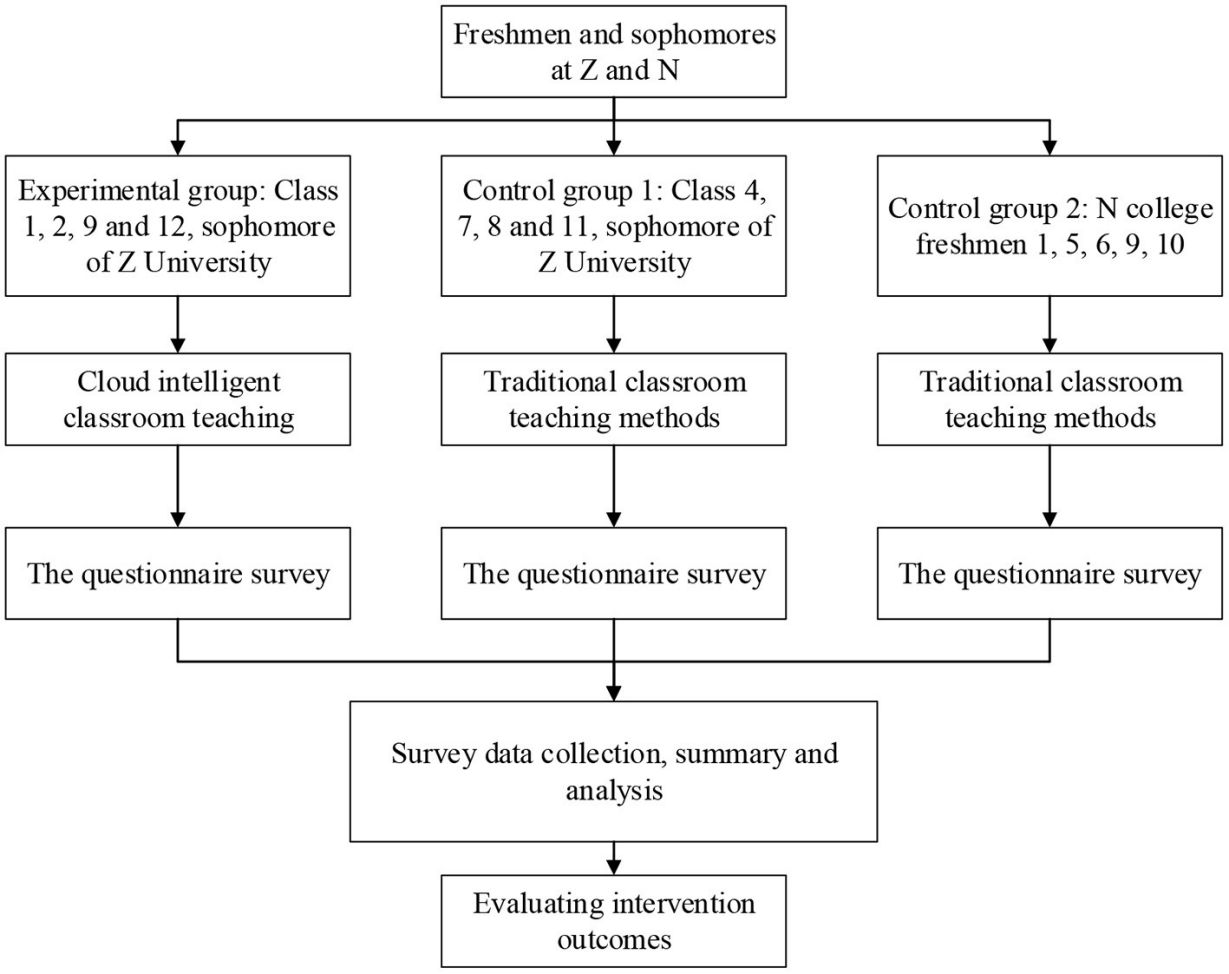
Research ideas and process.

Based on the theories and the current situation of the construction of the curriculum of mental health education, the cloud intelligent classroom of mental health education in the colleges and universities is constructed and the attitudes of the students toward the curriculum are explored through a questionnaire survey. Then, the experience of the students toward the course is tracked and investigated and experts are invited to observe and analyze the classroom records, evaluating the learning effect, and goal achievement. Finally, based on the practical experience and survey results, the research conclusion is drawn. The overall research process is shown in Figure 3. The subjects are divided into three groups, of which the experimental group takes the course in the cloud intelligent classrooms, while the other two control groups take the course in the traditional classrooms. The achievements are compared between the experimental groups and the control groups.

#### Research Subjects

The sophomores of Z University and the freshmen of N University in a city are selected as the research subjects. Since N University only offers freshmen courses for mental health education, this practical research is only carried out among the freshmen.

Among the sophomore of Z University, classes 1, 2, 9, and 12 are the experimental group, while the classes 4, 7, 8, and 11 are the control group 1, ensuring the consistency of each group as far as possible.

The freshmen in N University are randomly selected as control group 2.

#### Research Tools

(1) School-based teaching materials of the mental health education in the cloud intelligent classroom are “navigation in the sea of hearts” (for the freshmen and the sophomores).

The teaching process of this textbook mainly focuses on the mental health of the students, interpersonal communication, emotional regulation, and career planning. There are 10 class hours for the freshmen and five class hours for the sophomores, with a total of 15 class hours. The textbook is designed to include rich digital resources. At the beginning of the design, it attaches great importance to the personalized needs of the students and autonomous learning, which have a very significant effect on arousing the interest of the college students in learning and improving their psychological states.

(2) Questionnaire on the curriculum of the mental health education (questionnaire of the students).

The design of the questionnaire is inspired by some previous studies on the cloud intelligent classrooms and combined with the personality characteristics of the music majors to effectively measure the attitudes of the students toward the curriculum design of mental health education (Lei, 2019; Li, 2019; Min, 2019). Finally, goal achievement, student participation, class willingness, evaluation tendency, and the satisfaction of the students are selected as the measurement standards for the implementation effect of the curriculum design. The questionnaire is revised and completed based on the results of the previous interviews and the joint efforts of the full-time mental health education teachers, students, and experts in the field, which provide a guarantee for its effectiveness and professionalism. The reliability of each dimension of the questionnaire is above 0.7, which proves its good reliability and validity and can be used as a reference. There are 24 questions in the questionnaire, among which questions numbered 1–7 are used to investigate the achievements of the students in mental health education, questions numbered 8–12 are used to investigate the participation of the students, questions numbered 13–16 are used to investigate the willingness of the students to attend class, questions numbered 17–19 are used to investigate the evaluation tendency of the students, and questions numbered 20–24 are used to investigate the satisfaction of the students within the cloud intelligent classroom. The higher the scores, the more positive their attitudes toward the cloud intelligent classroom.

After the course ends, the questionnaire survey is conducted. 400 questionnaires are issued by Z University and 350 valid questionnaires are recovered, with a recovery rate of 87.5%. In the same week, the questionnaire survey on the control group 2 in N University is completed, which is jointly conducted by the researchers and teachers of the University. 220 questionnaires are distributed in N university and 182 are valid questionnaires, with a recovery rate of 82.73%.

### Educational Practice

#### Practical Mode

Posttests are carried out on control group 1, control group 2, and the experimental group (Table 1).

**Table 1:** Educational plan.

**Groups**	**Scope**	**Process**	**Post-test**
The experimental group	Z University	Teaching in the cloud intelligent classroom	Questionnaire on the curriculum of mental health education
Control group 1	Z University	Traditional classroom teaching	Questionnaire on the curriculum of mental health education
Control group 2	N University	Traditional classroom teaching	Questionnaire on the curriculum of mental health education

The specific differences in the process of teaching practice are presented in Table 2:

**Table 2:** Educational practice program.

	**Teaching in the cloud intelligent classroom**	**Teaching in the traditional classroom**
Lecture location	Special teaching in the cloud intelligent classroom	Classrooms
Discussion methods	Using teaching software to submit and display the results	Discussing and sharing
Investigation methods	Logging in and taking part in the survey	An anonymous survey
Classroom reflection	Taking a video in the teaching process	After class, teachers recall and write teaching postscript

*The cloud intelligent classroom has a tablet (one for each student or one for each group, which depends on the actual situation), network, touch screen TV, and network teaching software*.

#### Practical Process

The experiment is divided into four stages:

The first stage (2019.7–2019.12):
  (1) Determine the research ideas and select the research tools; (2) Determine the teaching materials and write the syllabus of the mental health education in the traditional classroom and the cloud intelligent classroom and also prepare the hardware and software debugging for the terminal teaching in advance; (3) Write a specific teaching plan and report to the relevant leaders for review; and (4) Communicate with the teachers who work on the mental health education at N University, share the course resources and teaching plans, and unify the teaching theme in the preparation stage.
The second stage (2020.3–2020.6):
  (1) The researcher setup the experimental group and the control group 1 in Z University for mental health education and the mental health education in N University is opened among the freshman and the students are the members of control group 2. The course focuses on the five topics: stress adjustment, heterosexual communication, interpersonal relationship, learning planning, and career development; (2) The experimental group and the control group only have differences in the teaching mode, while the teaching content and teaching time are the same as far as possible; and (3) A questionnaire survey is conducted on the experimental group, control group 1, and control group 2.
The third stage (2020.6-2020.8):
  (1) The data are analyzed and the results of the questionnaire survey are obtained and (2) Interview the teachers who teach the mental health courses to know about the current situation of the mental health education in the colleges and universities in N University.
The fourth stage (2020.10-2021.5), also known as the practice feedback stage:
  (1) The class and the students are followed-up to evaluate the teaching effect; (2) Invite the education experts to assess the classroom record and the teaching effect and invite the teachers to evaluate the course; and (3) Sort out the relevant data, so that the test process and results can be fully presented.

### Scheme and Control of the Independent Variables

#### Intervention Program

The teaching content is the textbook “*Xinhai navigation*” compiled by the universities. The textbook is based on the template of mental health education and its content is adjusted and modified several times according to the actual situation of the regions and universities. The contents of the study are as follows in Table 3:

**Table 3:** Teaching the topics and overview of the digital school-based course of mental health education.

**Times**	**Theme**	**Contents**
1	Go with pressure.	Properly handle the fatigue and anxiety of the postgraduate entrance examination and job hunting, and learn to face the increasing pressure of life and study.
2	It's not easy to say, love.	Examine the inner heart, treat the intimate relationship rationally, and learn to be kind to others and yourself.
3	Conflict brings growth—effective communication.	Master expression skills, learn to listen to others and skillfully resolve interpersonal conflicts.
4	Graduation is a small matter.	When graduation is coming, psychological construction should be conducted. A moderate goal should be set, and students should learn to plan life in a pressure environment.
5	On the boat of career—career planning guidance	A career can provide a variety of possibilities for life development. Students should recognize their own needs, and choose the path suitable for their personality development.

#### Control of the Independent Variables

(1) The course themes and teaching plans of Z University and N University should be consistent as far as possible and the teaching time is from March to June 2020.

(2) Control the other irrelevant variables. In the teaching process, teachers will not tell the students about the purpose of research in advance, so that the students can keep in a natural state.

## Data Analysis and Test Results

### Questionnaire Survey

1. The questionnaire results are statistically analyzed and the positive distribution test is carried out by the SPSS22. The data are expressed in mean ± SD, namely $\bar{x}\pm s$, as shown in Table 4.
2. The chi-squared test and *t*-test are used to test the results of the questionnaire.

**Table 4:** Overall situation of the questionnaire survey.

**Items**	**Groups**	**Totally agree**		**Agree**		**Disagree**		**Totally disagree**		***x* ± *s***
		**Frequency**	**%**	**Frequency**	**%**	**Frequency**	**%**	**Frequency**	**%**	**Frequency**
Help to achieve the goal	Experimental group	55	30.56	63	35	31	17.22	30	16.67	2.78 ± 1.05
	Control group 1	39	22.94	47	27.65	44	25.88	40	23.53	2.5 ± 1.09
	Control group 2	41	22.53	53	29.12	58	31.87	40	21.98	2.63 ± 1.08
Help to improve students' participation	Experimental group	73	40.56	58	32.22	28	15.56	21	11.67	3.02 ± 1.01
	Control group 1	26	15.29	63	37.06	62	36.47	19	11.18	2.56 ± 0.88
	Control group 2	29	15.93	58	31.87	73	40.11	22	12.09	2.52 ± 0.90
Help to improve the willingness to attend class	Experimental group	74	41.11	56	31.11	30	16.67	20	11.11	3.02 ± 1.01
	Control group 1	35	20.59	42	24.71	79	46.47	14	8.24	2.58 ± 0.91
	Control group 2	32	17.58	53	29.12	89	48.90	8	4.40	2.60 ± 0.82
Help to improve the evaluation tendency	Experimental group	80	44.44	67	37.22	19	10.56	14	7.78	3.18 ± 0.91
	Control group 1	19	11.18	37	21.76	55	32.35	59	34.71	2.09 ± 1.00
	Control group 2	25	13.74	38	20.88	66	36.26	53	29.12	2.19 ± 1.01
Help to improve students' satisfaction	Experimental group	59	32.78	60	33.33	30	16.67	31	17.22	2.81 ± 1.07
	Control group 1	44	25.88	52	30.59	35	20.59	39	22.94	2.59 ± 1.10
	Control group 2	55	30.22	45	24.72	47	25.82	35	19.23	2.66 ± 1.10

### The Chi-Squared Test on the Gender and Grade of Each Experimental Group

**Figure 4:**
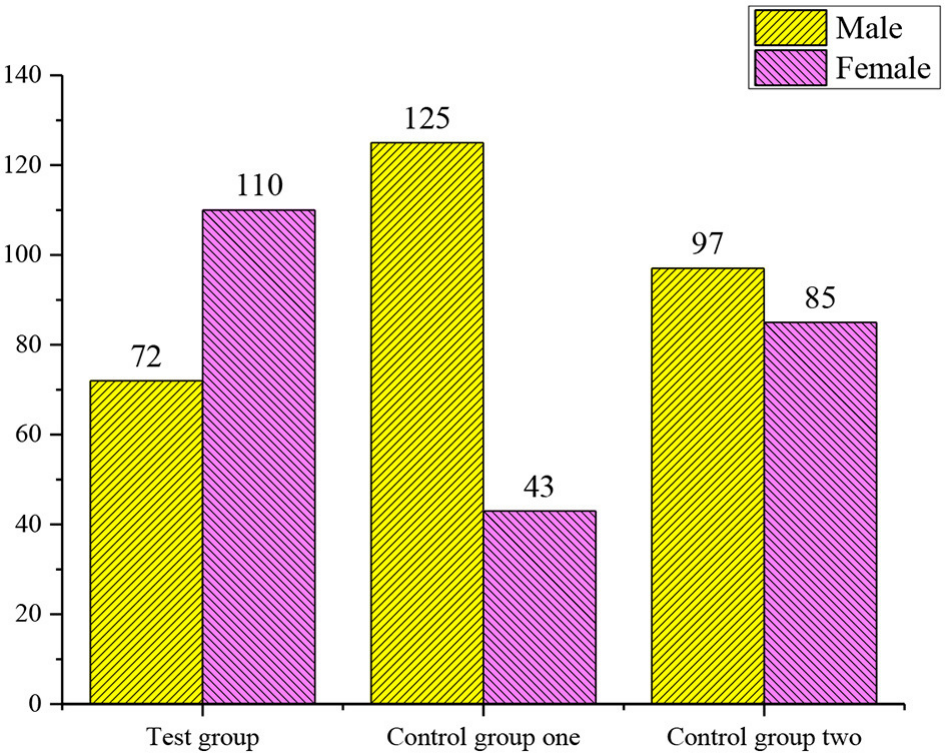
The chi-squared test of the gender among the experimental group, control group 1, and control group 2. χ^2^ = 0, *p* = 1.00 > 0.05. There is no significant difference in the gender among the groups.

**Figure 5:**
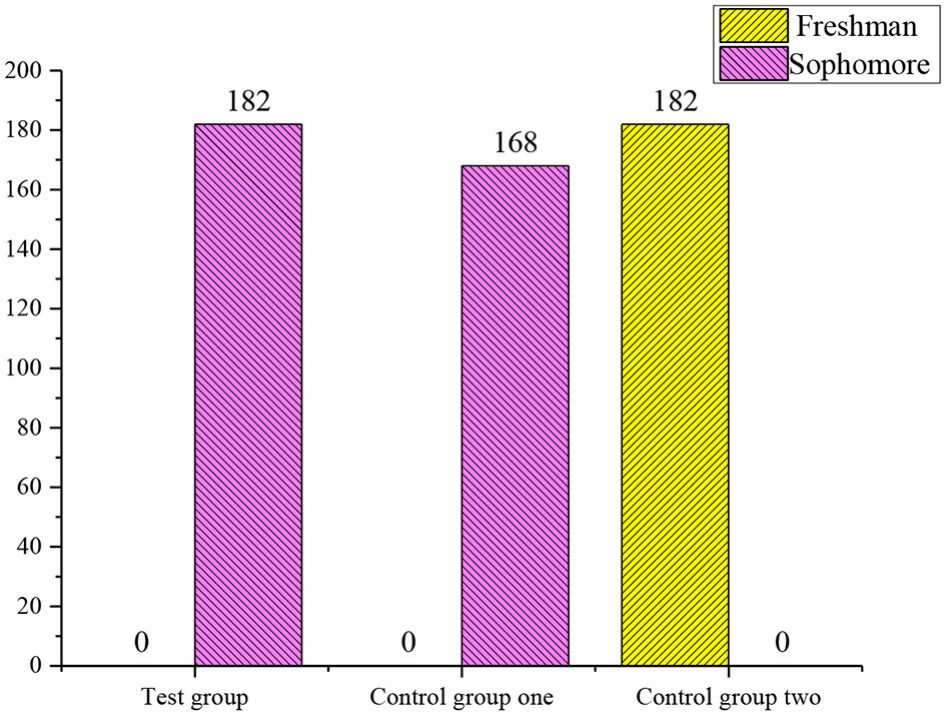
The chi-squared test on the gender of the experimental group, control group 1, and control group 2. χ^2^ = 0.3, *p* = 0.233 > 0.05. There is no significant difference in the grade variables among the three groups.

The above results show that the influence of gender and grade on the final results can be excluded (Figures 4, 5).

### Reliability of All the Dimensions of the Questionnaire

**Figure 6:**
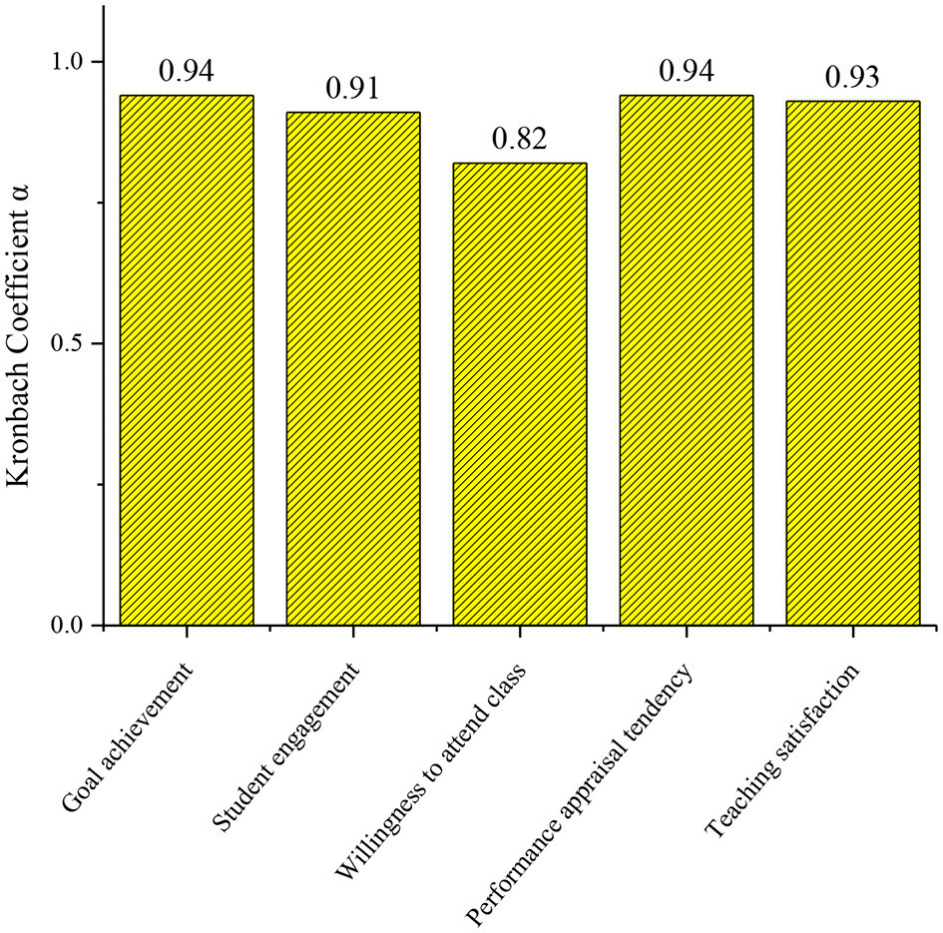
Reliability of each dimension of the questionnaire.

Figure 6 shows that the reliability of the questionnaire is good. In particular, the reliability coefficient α of the dimensions of goal achievement, teaching satisfaction, student engagement, and performance appraisal tendency is more than 0.9. The reliability coefficient of willingness to attend class is relatively low, but it also reaches 0.8. Overall, the results of this study are reliable.

### Goal Achievement of the Mental Health Education Based on the Cloud Intelligent Classrooms

**Figure 7:**
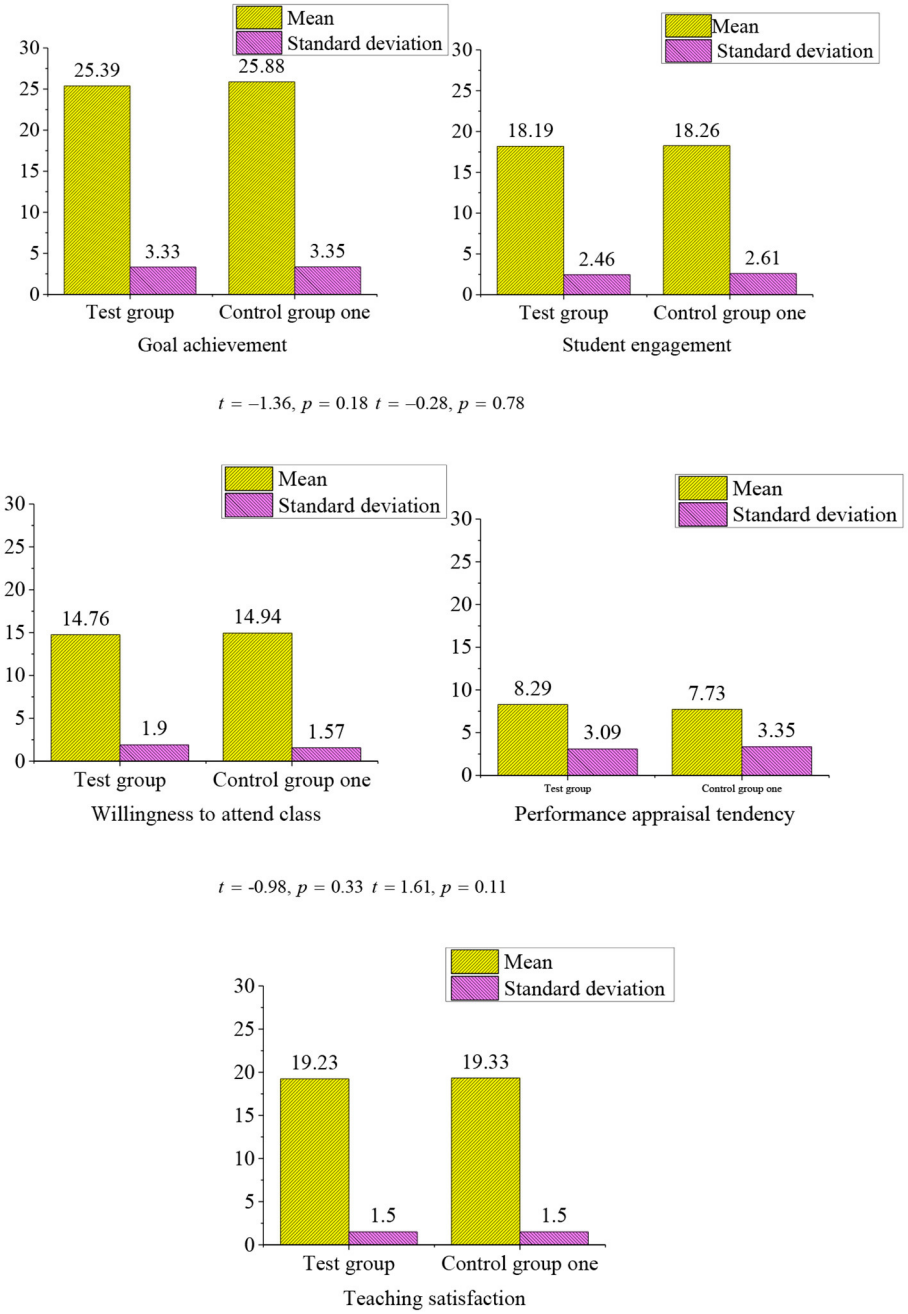
The *t*-test results of the independent samples of the experimental group and control group 1 (experimental group, *n* = 182, control group 1, *nl* = 168).

Figure 7 shows that the *p*-value is less than 0.05, which is significant. The test results of the experimental group and control group 1 indicate that the attitude of the students toward mental health education is relatively stable and is not affected by the changes in the form of courses after 3 months of mental health education is carried out in University Z. The cloud intelligent classroom is still in the development and exploration stage and both the teachers and students need a period to adapt and familiar with it. In addition, it is also related to the lack of attention to mental health education and the lack of corresponding examination-oriented assessment.

In terms of mean, the higher is the mean, the more is positive the attitude tendency of the students. The comparison shows that most students still prefer the traditional teaching mode of mental health education. In the dimension of performance appraisal tendency, students who receive mental health education in the cloud intelligent classroom have a higher degree of love for their teachers and they are more respected for the evaluation methods of learning. Due to the addition of human-computer interaction, the mental needs of college students are expanded (Brenda Happell et al., 2019). For the researchers, more attention should be given to the features and more care should be given to the students who are not paid attention to their study in the class instead of the top students in the traditional classroom, so that all the students can be treated equally (Danesh et al., 2019; Petchamé et al., 2021).

The recall of the course influences the learning effect of the course. Through the sampling survey of the experimental group and control group 1, the number of students who can recall the three or more subjects (a total of five courses) is called the effective number.

The Table 5 shows that the class that uses the cloud intelligent classroom to take the mental health education can acquire and memorize more knowledge. This is because the special classroom can leave more impression on the students compared to the traditional classroom. In addition, the teaching mode of human-computer interaction makes the engagement of students in the classroom effectively improved. In the traditional classroom, the students may distract from learning in the classroom and the traditional mode of speaking and feedback sharing is popular, so it is difficult to arouse the enthusiasm for study in the students.

**Table 5:** Percentage of the students correctly recalling the three or more subjects.

	**A class in the experimental group**	**A class in the control group**
Percentage of correct recalls	33.2%	13.4%

In conclusion, based on the above practice and investigation, the curriculum construction of the cloud intelligent classrooms is more impressive and influential than the traditional education classroom on the music majors and the evaluation of the student toward the teaching quality of the course is also higher.

### Assessment of Mental Health Education in Cloud Intelligent Classrooms by the Experts

Three teachers with senior professional titles or above are invited to review the classroom records (grade A 100–90, grade B 89–75, grade C 74–60, and grade D below 60).

**Figure 8:**
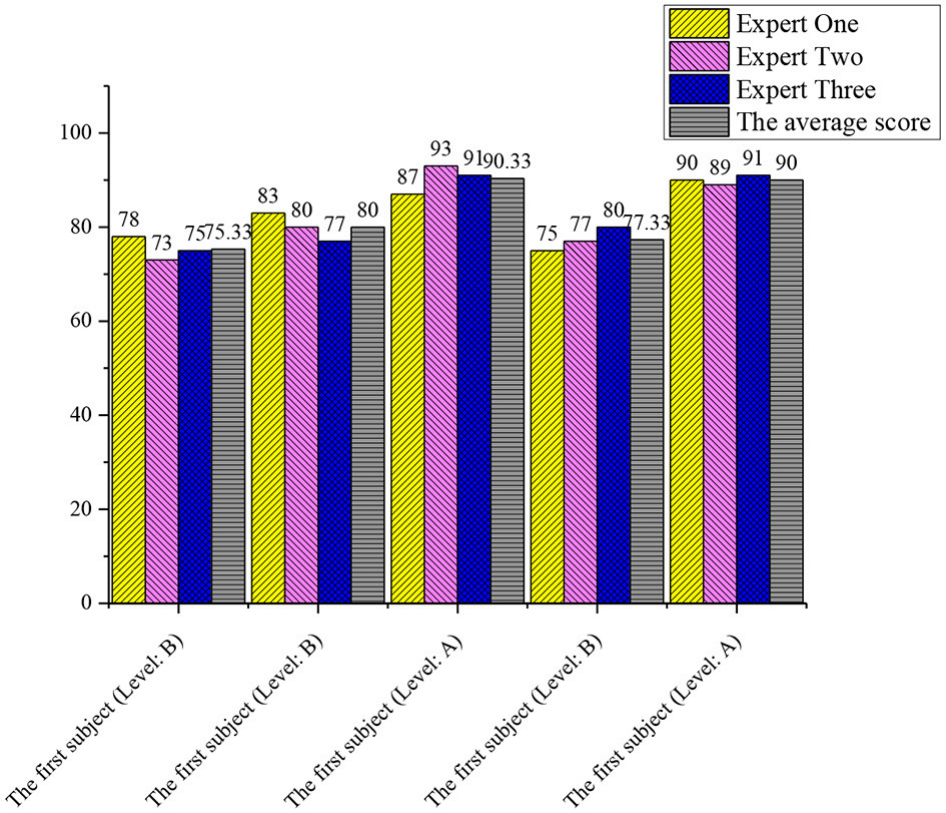
Experts scoring the records in the cloud intelligent classrooms.

According to the score of the record assessed by the experts (Figure 8), it is found that the mental health education in the cloud intelligent classroom meets the teaching requirements of the university, which can help the universities and teachers to achieve the expected teaching objectives and guide the students to insight into the deep knowledge. Experts believe that this is an innovative teaching mode and has a broad development space (Table 6).

**Table 6:** Assessment of the experts of the classroom observation.

**Items**	**Expert 1**	**Expert 2**	**Expert 3**
Student oriented	Excellent	Good	Excellent
Taking learning as the mainline	Good	Common	Good
Thinking- training intensity	Ordinary	Ordinary	Ordinary
Students' active engagement	Excellent	Good	Excellent
Achievement of teaching objectives	Good	Good	Excellent

Based on the results of the classroom observation, it is found that mental health education in the cloud intelligent classroom is student-centered and the students in the classroom can engage actively. This teaching mode based on digital technology is in line with the mental expectations of the students, helping teachers to achieve the teaching objectives. But at the same time, there are still shortcomings in the talent training in the cloud intelligent classroom, which distract the attention of the student from learning in certain situations. This requires that the teachers should adjust their teaching methods according to the characteristics of the cloud intelligent classrooms when designing the teaching plans (Happell et al., 2019; Hayas et al., 2019; Wise et al., 2019).

### Interschool Differences in Mental Health Education in Colleges and Universities

**Figure 9:**
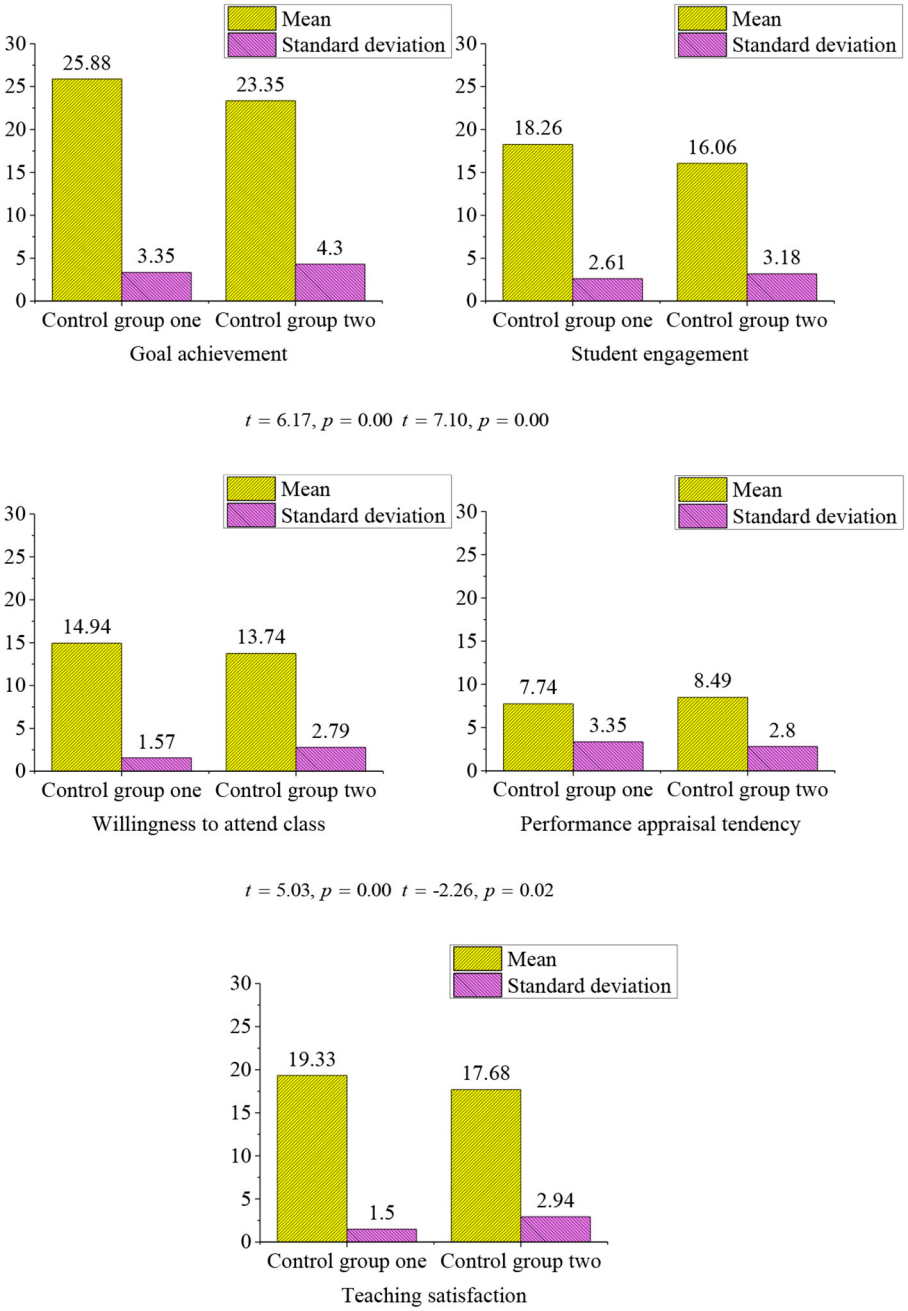
The *t*-test of the control group 1 and control group 2 (control group 1, *n1* = 168, control group 2, *n2* = 182).

Since the two control groups are taught in the traditional classrooms, there are significant differences in the learning effect (Figure 9). This may be because the different universities have significant differences in the above dimensions of mental health education, which has a great impact on the realization of the teaching objectives. The test results show that Z University is more positive in the evaluation of mental health education, while the students in N university are more willing to accept the evaluation of teachers, which are closely related to the pressure of the performance in Z University.

## Discussion

The above investigation and practical research are closely related to the actual work of mental health education in colleges and universities. The educational action research method is adopted and the qualitative analysis and quantitative analysis are combined to conduct the practice research based on compiling the teaching materials. The results of the questionnaire survey and tracking survey show that cloud intelligence courses can achieve the teaching objectives of mental health education. Evaluation by the experts and teachers shows that this course is a bold attempt and achieves a good effect. It can optimize the process of mental health education. It expands the connotation of digital courses and pursues the unity of “form” and “content” while updating the means and methods of mental health education for college students. Based on the above investigations, the following conclusions are drawn:

1. In the long term, mental health education in the cloud intelligent classroom can improve the teaching quality of the mental health education of the music majors in colleges and universities.
2. Cloud intelligence courses of mental health education can help to achieve the teaching objectives.
3. Mental health education in the cloud intelligent classroom is helpful to enhance the learning effect of mental health education.
4. Based on the results of the mental health education in N University, it is proved that there are differences in the mental health education in the different regions and universities. Therefore, it is imperative to promote mental health education in the cloud intelligent classroom.

In comparison to the previous study, it is found that the research results confirm the practice results of the mental health education by Lattie EG based on the Claroline platform in high school. In the auxiliary teaching based on the digital platform, the students can be more concentrated in the teaching activities than the traditional mental health education and the diversified multimedia attracts more students. Feedback of the students after the class is more positive and they are willing to spend time on precipitation, digestion, and presentation (Lattie et al., 2019). This study also echoes the mental health education based on the Moodle platform developed by Pine. Digital courses make up for the one-dimensional nature of the traditional curriculum discussion and communication. They make use of open and interactive teaching techniques to facilitate communication and cooperation, resource sharing, innovative education management, and learning modes among students, improving the efficiency of the curriculum design (Pine, 2020).

## Conclusion

Based on the combination of the quantitative and qualitative methods and deep learning, the research on the teaching mode and the ideas in the cloud intelligent classrooms are studied. Through the results of the questionnaire survey and expert assessment, it can be found that mental health education in the cloud intelligent classroom is an innovation for the traditional teaching mode and has a good learning effect, which promotes the realization of the teaching objectives.

In mental health education, the relevant personnel should pay special attention to the following aspects. In terms of the teaching operation, both the teachers and students should be familiar with the teaching environment of the cloud intelligent classrooms in advance. It is suggested that class hours should be setup so that the students can have a relatively clear understanding of the operation of the digital teaching software and terminal equipment before class. It can effectively distinguish the teaching instruction proposed by the lecturer and play a role in maintaining the classroom instructions. As for the feedback, the teachers should feedback timely to overcome the defects of the software system. For students, feedback can enhance the interaction between the teachers and students, timely discussing the problems encountered in the teaching process. Concerning curriculum evaluation, different evaluation methods and models should be studied and updated. The traditional curriculum evaluation mode is mainly teacher-oriented, while cloud intelligent classroom is student-oriented, reflecting the attitude of the students toward the course and homework after class. About information preservation, the cloud intelligent classroom of mental health education in colleges and universities should produce a lot of data in the teaching process. Teachers can screen and classify these data after class and establish the databases to ensure a quick search for useful information later.

In addition, as for the teaching tools, the current learning platforms and Apps mainly concentrate on how to facilitate the workload of the teachers, but the essence of education is to promote the development of the students. Therefore, how to deeply excavate the influence of the learning platforms and Apps on the students will be the focus of the follow-up study and it needs to be accumulated in the teaching practice.

## Data Availability Statement

The raw data supporting the conclusions of this article will be made available by the authors, without undue reservation.

## Ethics Statement

The studies involving human participants were reviewed and approved by Gannan Normal University Ethics Committee. The patients/participants provided their written informed consent to participate in this study. Written informed consent was obtained from the individual(s) for the publication of any potentially identifiable images or data included in this article.

## Author Contributions

All authors listed have made a substantial, direct and intellectual contribution to the work, and approved it for publication.

## Conflict of Interest

The authors declare that the research was conducted in the absence of any commercial or financial relationships that could be construed as a potential conflict of interest.

## Publisher's Note

All claims expressed in this article are solely those of the authors and do not necessarily represent those of their affiliated organizations, or those of the publisher, the editors and the reviewers. Any product that may be evaluated in this article, or claim that may be made by its manufacturer, is not guaranteed or endorsed by the publisher.
