# Supplementary material for: Applying the Cloud Intelligent Classroom to the Music Curriculum Design of the Mental Health Education
Source: Front Psychol. 2021 Nov 16;12:729213. doi: 10.3389/fpsyg.2021.729213 (PMC8637775; doi:10.3389/fpsyg.2021.729213)
Supplement: Supplementary file 1 [file Data_Sheet_1.docx]

**Appendix**

**Questionnaire on the curriculum of mental health education**

Dear students,

This questionnaire survey is conducted to improve the teaching quality of mental health education and provide students with better services for their mental health. Please take a few minutes to and answer the questionnaire. This questionnaire is anonymous. All data are only used for statistical analysis. There are no right or wrong options. Please complete the questionnaire according to the actual situation. Thank you for your cooperation!

Class: Gender:

Read the following questions. Please tick or underline the options according to your opinions.

1. Mental health education helps us prevent mental problems.

A. Totally agree B. agree C. disagree D. totally disagree

2. Mental health education provides us with a lot of mental health knowledge

A. Totally agree B. agree C. disagree D. totally disagree

3. Mental health education makes us establish the consciousness of mental health care

A. Totally agree B. agree C. disagree D. totally disagree

4. Mental health education makes us relax in activities

A. Totally agree B. agree C. disagree D. totally disagree

5. Mental health education improves my ability to control my emotions and helps me to know how to get along with others.

A. Totally agree B. agree C. disagree D. totally disagree

6. Mental health education promotes the formation of our good attitude.

A. Totally agree B. agree C. disagree D. totally disagree

7. In mental health class, I often get some insights by myself.

A. Totally agree B. agree C. disagree D. totally disagree

8. In the mental health class, I actively participated in the game activities organized by the teacher.

A. Totally agree B. agree C. disagree D. totally disagree

9. In mental health class, I actively participate in case discussion

A. Totally agree B. agree C. disagree D. totally disagree

10. In mental health class, I actively participate in role-playing activities.

A. Totally agree B. agree C. disagree D. totally disagree

11. In mental health class, I usually listen carefully.

A. Totally agree B. agree C. disagree D. totally disagree

12. In the mental health class, I have the opportunity to express my opinions on the content and teaching methods of the course.

A. Totally agree B. agree C. disagree D. totally disagree

13. I look forward to taking every mental health class.

A. Totally agree B. agree C. disagree D. totally disagree

14. I hope there will be more psychological counseling in the class meeting.

A. Totally agree B. agree C. disagree D. totally disagree

15. I hope to increase the teaching time of mental health classes.

A. Totally agree B. agree C. disagree D. totally disagree

16. I hope there are more mental health courses in the ideological and political classes.

A. Totally agree B. agree C. disagree D. totally disagree

17. I hope the teacher can evaluate our performances in mental health classes.

A. Totally agree B. agree C. disagree D. totally disagree

18. I hope the teacher can evaluate our

grades according to our performance in mental health class

A. Totally agree B. agree C. disagree D. totally disagree

19. I hope the teacher can evaluate our grades according to our summary after mental health classes.

A. Totally agree B. agree C. disagree D. totally disagree

20. I am very satisfied with the teacher's teaching attitude in mental health classes.

A. Totally agree B. agree C. disagree D. totally disagree

21. I'm very satisfied with the content of mental health classes.

A. Totally agree B. agree C. disagree D. totally disagree

22. I am very satisfied with the method used in mental health classes.

A. Totally agree B. agree C. disagree

D. totally disagree

23. I am very satisfied with the teaching quality of mental health teachers

A. Totally agree B. agree C. disagree D. totally disagree

24. I am very satisfied with my learning activities in mental health classes.

A. Totally agree B. agree C. disagree D. totally disagree
